# Chromosome-level assembly of the *Glechoma longituba* genome

**DOI:** 10.3389/fpls.2025.1597825

**Published:** 2025-09-25

**Authors:** Yubang Gao, ChenQi Zhao, Dongdong Yang

**Affiliations:** ^1^ School of Life Sciences, Nanyang Normal University, Nanyang, Henan, China; ^2^ The First Affiliated Hospital, Shihezi University, Shihezi, China

**Keywords:** genome assemble, Chinese herbal medicine, *Glechoma longituba*, nanopore sequence, Hi-C sequence

## 1 Introduction

*Glechoma longituba*, a perennial species in the Lamiaceae family widely distributed across Eurasia, is commonly known as long-leaved ground ivy. It typically grows in moist, fertile environments such as forest edges, stream banks, or valley grasslands (Zhou et al., 2021). *G. longituba*, commonly known as Huoxuedan in China, is a traditional Chinese medicinal herb and an edible wild plant in the mountainous regions of southern Shaanxi Province. *G. longituba* contains a variety of pharmacologically active compounds, including terpenoids, steroids, flavonoids, polyphenols, alkaloids, and fatty acids (Ouyang et al., 2019a, 2019; ZhU, 2013; Zhu et al., 2013; Zhang et al., 2006; Zhi-bin et al., 2008). Extracts of *G. longituba* have shown potential in preventing and treating kidney stones (Yang et al., 2011; Luo et al., 2019), as well as possessing anti-inflammatory, analgesic (Luo et al., 2020; Chou et al., 2019), antioxidant (Xian and Xie, 2014; Liu et al., 2016), anti-cancer, and antiviral properties (Ouyang et al., 2019a, 2019), and in reducing blood sugar levels (Yang et al., 2021).

Despite its pharmacological significance, *G. longituba* lacks comprehensive genomic resources. The *G. longituba* genome assembly will establish its distinct advantages for advancing research within the Lamiaceae family. Unlike the well-characterized genomes of economically important relatives (e.g.Salvia), *G. longituba* possesses unique biological features—specifically, its aggressive stoloniferous growth enabling rapid clonal colonization and significant shade tolerance. We will exploit its high-quality, contiguous genome assembly to identify the genetic determinants underlying these key adaptive traits, which are largely unexplored in core Lamiaceae crops. Furthermore, *G. longituba* is a rich source of bioactive terpenoids and flavonoids, some exhibiting unique profiles compared to close relatives. To address this gap, we present the first chromosome-scale genome assembly using an integrated approach combining Oxford Nanopore Technologies(ONT), Hi-C chromatin conformation capture, and short-read polishing. This high-quality reference genome enables systematic exploration of its metabolic biosynthesis, polyploidization history, and evolutionary relationships within Lamiaceae.

## 2 Materials and methods

### 2.1 Material collection and genomic DNA sequencing

Plants used for genomic sequencing were cultivated under laboratory conditions of 25°C, 3000 lx, and a 16-hour light: 8-hour dark photoperiod. High Molecular Weight (HMW) DNA was extracted for subsequent library construction using the Qiagen MagAttract HMW DNA Mini Kit, following the manufacturer’s protocol. Purified DNA was prepared using magnetic beads. Sequencing adapters from the SQK-LSK109 kit were then ligated to the purified product. The constructed DNA library was precisely quantified using Qubit. Following library preparation, a defined concentration and volume of the DNA library was loaded onto the Flow Cell. The Flow Cell was subsequently transferred to the Oxford Nanopore PromethION sequencer for real-time single-molecule sequencing. Basecalling was performed using Dorado v0.8.3 with the dna_r9.4.1_e8_hac@v3.3 model, followed by correction of the sequencing data using Dorado.

Short-read sequencing was performed on the DNBSEQ-T7 platform. Short reads were utilized for genomic survey analysis, including genome size estimation, heterozygosity, repeat content, and for correcting long-read sequencing assemblies. Long reads were used for contig-level genome assembly.

### 2.2 Hi-C library construction and sequencing

To determine the order and orientation of contigs, chromosome conformation of the genome was captured. Plant leaf samples were ground and cross-linked with 2% formaldehyde solution in nuclear separation buffer at room temperature for 10 minutes. Fixed cells were digested with the MboI enzyme. Digestion was followed by cell lysis, incubation, labeling DNA ends with biotin-14-dCTP, and ligating blunt-ended cross-linked fragments. The Hi-C library underwent 12–14 PCR cycles of amplification and was sequenced on the DNBSEQ-T7 platform.

### 2.3 Genome survey

Genomic features were estimated based on short reads. The original sequences were trimmed using the fastp software (Chen et al., 2018) version 0.20.1 with default parameters. K-mer distribution histograms were calculated with jellyfish (Marçais and Kingsford, 2011) version 2.3.0, with parameters “-m 21 -s 50G -t 48”. Genome size, heterozygosity, and repeat content were estimated using GenomeScope 2.0 (Ranallo-Benavidez et al., 2020), with the parameters “-p 4 –kmer 21”.

### 2.4 Genome assembly and annotation

Long reads from ONT sequence were quality controlled and assembled into contigs using the “correct then assemble” strategy in nextDenovo (Hu et al., 2023) version 2.5.2, with parameters “read_cutoff = 1k, genome_size = 400m, pa_Correction = 4, sort_options = -m 20g -t 10, minimap2_options_raw = -t 10, Correction_options = -p 10, minimap2_options_cns = -t 10, nextgraph_options = -a 1”.Redundancies in the genome were removed using purge_haplotigs version 1.1.3 (Roach et al., 2018) with the parameter ‘-a 65’. Subsequently, the contigs were polished four rounds using Nextpolish version 1.4.1 (Hu et al., 2020) with default parameters guided by short-read data. The polished contigs were assembled into a chromosomal-level genome using Hi-C sequencing data. Low-quality reads and adapters from the Hi-C library were filtered using Trimmomatic (Bolger et al., 2014) version 0.39 with default parameters, followed by mapping to the assembled contigs using Juicer (Durand et al., 2016) version 1.5. Reads were grouped into chromosomes using 3D-DNA (Dudchenko et al., 2017) version 180922 with parameters ‘–editor_repeat_coverage = 40, -r 0’. Errors were manually adjusted in Juicebox version 2.16.00 (https://github.com/aidenlab/Juicebox). The original chromosomes were updated using the “run-asm-pipeline-post-review.sh” script from 3D-DNA. The CRAQ algorithm (Li et al., 2023) (version 1.0.9) was employed to calculate the Alignment Quality Index. This metric quantifies anomalies in clipped alignment segments, serving as an indicator of potential misjoins in genome assemblies. Genome assembly quality was assessed using BUSCO (Manni et al., 2021) v5.5.0. Finally, repetitive sequences were annotated using EDTA (Ou et al., 2019) version 2.0.1 with default parameters. Gene prediction was performed using BRAKER3 (Gabriel et al., 2023) version 3.0.8. Functional annotation was executed by blasting proteins against the SwissProt/NR/TAIR databases using diamond (Buchfink et al., 2015) version 2.0.14.152 with parameters: ‘–strand plus -k 1 –evalue 1e^-5^’.

### 2.5 Phylogenetic analysis

For phylogenomic reconstruction, protein sequences of 10 representative species were retrieved from public repositories: *Amborella trichopoda* (basal angiosperm), *Oryza sativa* (Poaceae), *Vitis vinifera* (Vitaceae), *Theobroma cacao* (Malvaceae), *Arabidopsis thaliana* (Brassicaceae), *Solanum lycopersicum* (Solanaceae), *Coffea canephora* (Rubiaceae), *Tectona grandis* (Lamiaceae), Leonurus japonicus (Lamiaceae), and Salvia miltiorrhiza (Lamiaceae). Orthologous gene clusters were identified using OrthoMCL with inflation parameter 1.5 and E-value cutoff 1e^-5^. A maximum-likelihood phylogeny was inferred from concatenated single-copy orthologs using FastTree2 under the JTT+CAT substitution model, with branch support evaluated by 1,000 Shimodaira-Hasegawa (SH) approximate likelihood ratio tests (α=0.05). Divergence time estimation was performed in r8s using penalized likelihood with three fossil calibrations: *A. thaliana*-*T. cacao*, *V. vinifera*-*A. trichopoda*, and *L. japonicus*-*T. grandis*. Gene family dynamics were analyzed through CAFE using a global birth-death rate (λ=0.002) and significance thresholds adjusted by the Benjamini-Hochberg false discovery rate (FDR <0.01).

## 3 Data

### 3.1 Genome assembly

**Figure 1:**
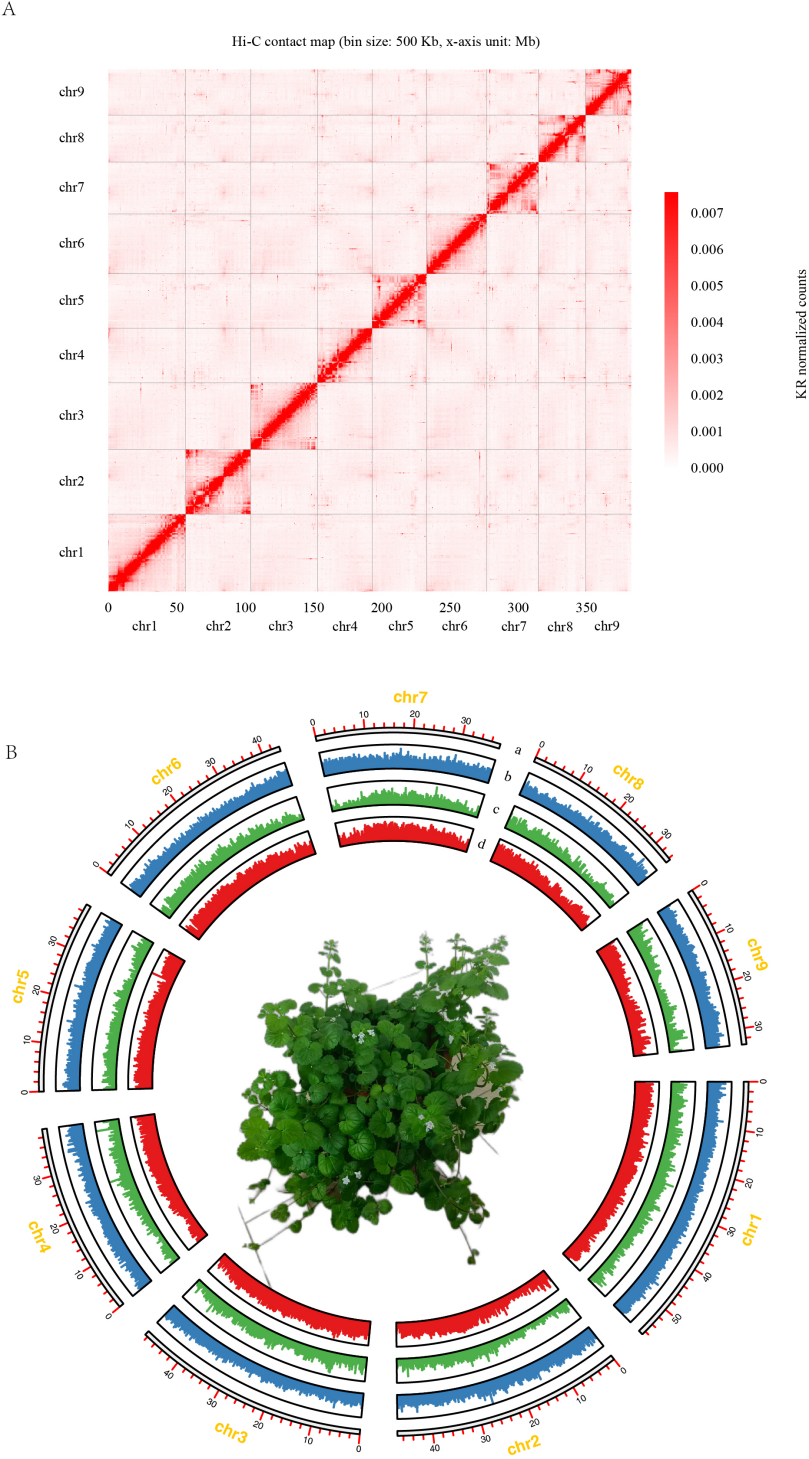
Chromosome-scale assembly of the *G. longituba* genome. **(A)** Contact map of *G. longituba* genome. **(B)**. Circos plot displaying the 12 chromosomes in the *G. longituba* genome. a. Length of each pseudochromosome (Mb). b. Distribution of repetitive sequences. c. Distribution of gene density. d. Distribution of the GC content. The center is the phenotype of *G. longituba* (The flower pot size was 15 cm).

The sequencing process yielded 49.40 Gb of clean short-read data and 50.26 Gb of long-read data (**Supplementary Table S1**). A total of 180 GB of clean data was generated, representing a 370.4-fold genome coverage (**Supplementary Table S1**). The estimated genome size is 396 MB. Previous studies have shown that *G. longituba* is tetraploid (Jang et al., 2016), with results indicating aabb (3.81%) >aaab (0.001%), suggesting an allopolyploid genome (**Supplementary Figure S1**). A total of 208 contigs were assembled into 9 chromosomes (**Figure 1A**). The largest chromosome measures 54.2 Mb, and the smallest is 31.7 Mb. Chromosomes were numbered in descending order of size. The anchored genome spans a total length of 390 Mb with an N50 of 37.8 Mb. The high-quality genome assembly of *G. longituba* was further validated by its LTR Assembly Index (LAI) of 13.13, surpassing the threshold for reference-grade genomes (LAI >10). The final assembly achieved 94% BUSCO completeness (embryophyta_odb10, **Supplementary Table S2**), with CRAQ quality score of 97.92 and contiguity metrics (N50 = 37.8 Mb, L50 = 5, N90 = 31.7 Mb, L50 = 9). Polyploid genome assembly can be conceptualized as the summation of multiple haplotype reconstruction problems, with computational complexity increasing significantly with higher ploidy levels. As an assembly study of an allotetraploid genome, this project successfully generated a collapsed genome assembly. This initial assembly can serve as a foundation for reconstructing complete subgenomes in future research. This robust assembly provides a reliable foundation for downstream evolutionary and functional analyses.

### 3.2 Gene prediction and gene annotation

The final genome assembly has a GC content of 35.84%. The genome comprises 52.13% repetitive sequences, with Type I Transposable Elements (TEs) constituting 26.41% and Type II TEs constituting 25.72%. A total of 28,437 protein-coding genes were identified, of which 26,508 have functional annotations (**Figure 1B**, **Supplementary Table S3**). A total of 1,060 non-coding RNAs (ncRNAs) were predicted, comprising 121 rRNAs, 96 miRNAs, 264 snRNAs, and 579 tRNAs.

### 3.3 Phylogenetic analysis of *G. longituba*

**Figure 2:**
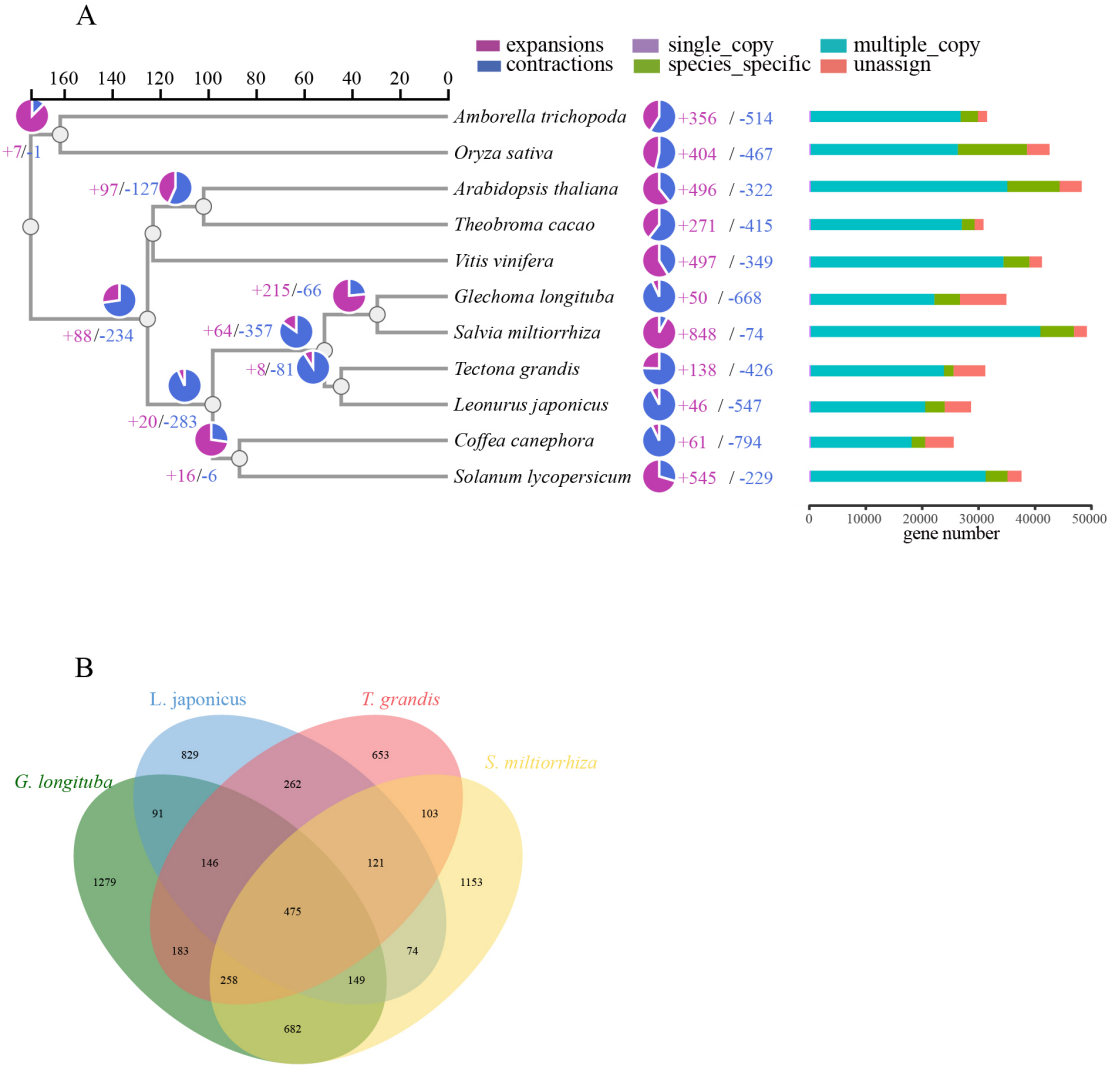
Evolutionary analysis of the (*G*) *longituba* genome. **(A)** A phylogenetic tree based on shared single-copy gene families, gene family expansions, and contractions among (*G*) *longituba* and ten other species. The bar chart on the right displays gene family clustering in (*G*) *longituba* and ten other plant species. **(B)** Venn Diagram Representation of Gene Family Overlaps and Specificities Among (*G*) *longituba*, *L. japonicus*, *T. grandis*, and *S. miltiorrhiza* in Labiatae.

A total of 401,514 proteins from 11 species were clustered, yielding 268 single-copy orthologs (**Supplementary Table S4**). A divergence time tree was constructed by incorporating known fossil calibration points (**Figure 2A**), which estimated the divergence time between *G. longitumba* and *S. miltiorrhiza* to be 25.99 million years ago (MYA). Gene family expansion and contraction analysis using the CAFE program revealed 50 contracted and 668 expanded gene families in the *G. longituba* genome. Comparative analysis of gene families among *G. longituba*, *L. japonicus*, *T. grandis*, and *S. miltiorrhiza* identified 475 conserved gene families and 1,279 species-specific gene families (**Figure 2B**). These genes are significantly enriched in biological processes such as terpenoid biosynthesis (e.g., monoterpenes and sesquiterpenes).

## Data Availability

The genomic short-read, long-read, and Hi-C data are available in the NCBI Sequence Read Archive (SRA) under accession numbers SRR28493072, SRR28493070, and SRR28493071, respectively. The assembled genome and annotation files are stored on Figshare at https://doi.org/10.6084/m9.figshare.25506331.v1 and the National Genomics Data Center (NGDC) with accession number GWHGEUA00000000.1.
