# Supplementary figures and images for: Chromosome-level assembly of the Glechoma longituba genome
Source: Front Plant Sci. 2025 Sep 25;16:1597825. doi: 10.3389/fpls.2025.1597825 (PMC12507912; doi:10.3389/fpls.2025.1597825)

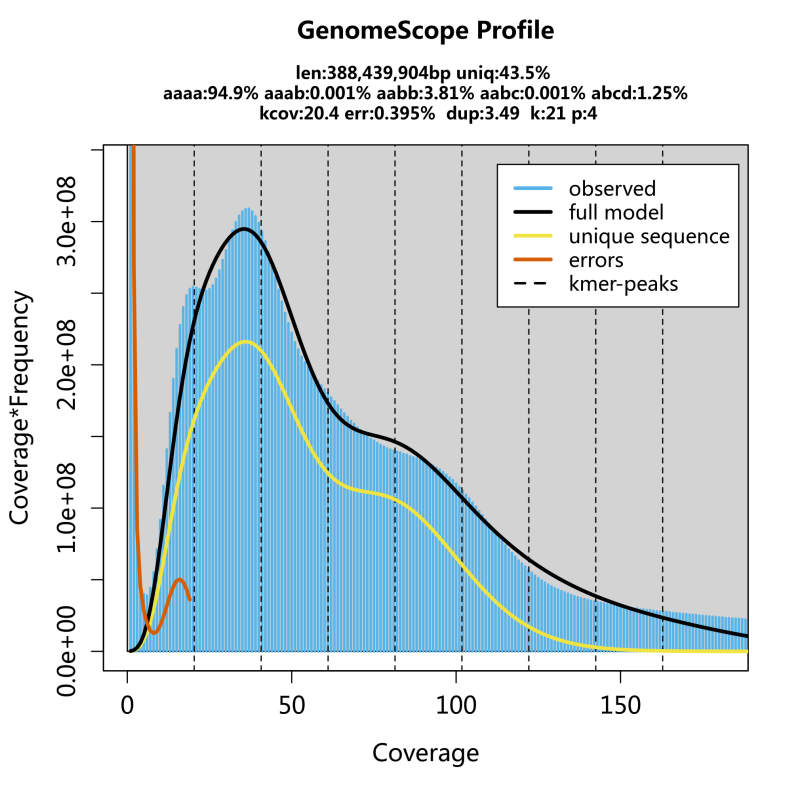


Fig. S1 Genome survey of *G. longituba*.

Supplement: Supplementary file 1 [file Supplementaryfile1.docx]
